# Performances, carcass characteristics, and economic benefit of yearling Hararghe highland rams fed diet containing concentrate mixtures and sugarcane bagasse or rice husk treated with *Trichoderma viride* and effective microorganisms

**DOI:** 10.1002/fsn3.3687

**Published:** 2023-09-15

**Authors:** Regasa Begna, Mengistu Urge, Tegene Negesse, Getachew Animut

**Affiliations:** ^1^ Department of Animal Science Mizan Tepi University Mizan Teferi Ethiopia; ^2^ School of Animal and Range Science Haramaya University Dire Dawa Ethiopia; ^3^ Schoolof Animal and Range Science Hawassa University Hawassa Ethiopia; ^4^ Agricultural Transformation Agency Addis Ababa Ethiopia

**Keywords:** biological treatment, carcass, feed conversion efficiency, profitability, sheep

## Abstract

The productivity of small ruminants in Ethiopia is low due to a shortage of feed supply throughout the year, both in terms of quality and quantity. This problem leads to the utilization of locally available lignocellulose by‐products, which encompass high cellulose, hemicellulose, and lignin. It is known that the nutritive value of these lignocellulose by‐products can be improved through biological, chemical, or a combination of both treatments This study was aimed at evaluating body weight change, carcass characteristics, and economic efficiency of rams fed a total mixed diet containing biologically treated rice husk (RH) or sugarcane bagasse (SCB). Thirty‐six sheep, weighing an average initial body weight of 18 ± 1.8 kg were used. Two feeds (SCB and RH) and three biological treatments (Control, *Trichoderma viride* [Tv], and effective microbes [EM]) were used with a randomized complete block design (RCBD) in 2 × 3 factorial arrangements. Ram fed on RH containing diets had higher dry matter intake (DMI) (g/h/day), DMI (% of BW), DMI (g/kg BW^0.75^), average daily gain (ADG) (106 vs. 53 g/day), and feed conversion efficiency (FCE) (0.107 vs. 0.076) than SCB containing diets. Ram fed diets containing biologically treated roughages had significantly higher (*p* < .05) DMI (g/h/day), DMI (% of BW), DMI (g/kg BW^0.75^), ADG, and FCE. However, no significant differences were observed between sheep fed diets containing EM and Tv in DMI (g/h/day), DMI (% of BW), DMI (g/kg BW^0.75^), ADG, and FCE. This study also revealed that significantly (*p* < .05) higher carcass weight, dressing percentage (DP), rib eye muscle area, total non‐carcass fat, and total edible offal components had been recorded for rams fed diets containing RH as roughage source related to rams fed diets containing SCB. While other parameters were unaffected by treatments, sheep fed diets containing biologically treated feed had a higher hot carcass weight plus DP. However, no significant (*p* > .05) difference was found between diets based on biologically treated roughage. Profitability analysis indicated that sheep fattening on a diet containing RH and by‐products treated with Tv and EM showed greater profitability than untreated SCB. The authors had concluded that fattening sheep on diets containing RH results in higher feed intake, better body weight gain, more carcasses and higher profits, but optimum inclusion levels need further research, for either treated or untreated SCB.

## Graphical abstract

This study was aimed to evaluate body weight change, carcass characteristics, and economic efficiency of rams fed total mixed diet containing biological treated rice husk (RH) or sugarcane bagasse (SCB). The results revealed that significantly (*p* < .05) higher carcass weight, dressing percentage (DP), rib eye muscle area, total non‐carcass fat, and total edible offal components had recorded for rams fed diets contained RH as roughage source related to rams fed diet contained SCB. While other parameters were unaffected by treatments, sheep fed diet containing biologically treated feed had higher hot carcass weight plus DP. Finally, we concluded that fattening sheep on diets containing RH yield more carcass and higher profits, but optimum inclusion levels need further research, particularly for SCB.

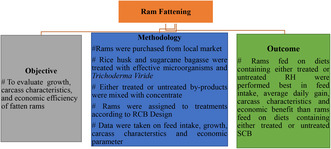

## 1 INTRODUCTION

Sheep in Ethiopia are the second most important species, with an estimated number of about 42.91 million (CSA,  2021b), and are mainly kept for meat production (Ewnetu et al., 2006). The annual national mutton production of the country is estimated at about 77,000 metric tons at a 30% offtake (Amha, 2008) and an average carcass weight of 10.1 kg, the second lowest among sub‐Saharan African countries (FAO, 2009). Although Ethiopia has high genetic diversity, their productivity is low, mainly because of an inadequate year round feed supply, both in terms of quality and quantity (Alemu & Merkel, 2008). Livestock in general and sheep in particular are nourished with natural pasture and crop residues, which are known to have low nutritional content, low digestibility, and low voluntary intake (Adugna, 2007).

Scarcity of animal feed in the developing world attracts the attention of researchers to the efficient utilization of lignocellulose as an agro‐industrial by‐product. Evidence indicate that about 53.2 million tons of lignocellulose agricultural by‐products are produced annually in Ethiopia (CSA, 2021a). Currently, about 4 million (Esayas et al., 2018) and 27,200 (CSA, 2016) tons of sugarcane bagasse (SCB) and rice husks (RHs) are produced annually, respectively. Cultivation of crops also generates a huge amount of fibrous crop residues, such as cereal straws, legume straws, and oil crop straws, which are usually fed to ruminants. The primary factors limiting the use of this lignocellulose are its low nutrient content, low digestibility, and low voluntary intake. Their low digestibility and voluntary intake are generally associated with high fiber content, composed mainly of 32%–40% cellulose, 14%–36% hemicelluloses, and 10%–18% lignin based on dry matter (DM) (Mussatto & Teixeira, 2010). In the last few decades, researchers have raised the need to improve nutritional value of dietary fiber for ruminants through overcoming the inherent barriers to rumen microbial fermentation (Sarnklong et al., 2010). This can be done by using physical, chemical, and biological treatment methods and their combinations (Preston, 2003).

The use of microorganisms and their enzymatic machinery to break down lignin and modify the structure of lignocellulose is defined as a biological treatment. According to Jalč (2002), the use of fungi and/or their enzymes that metabolize lignocellulose is a potential biological treatment to enhance the nutritional contents of agro‐industrial by‐products. Most research reports indicate that feeding ruminants with biologically treated roughage increases feed intake, improves digestibility, and increases feed conversion efficiency (FCE) compared to untreated (Abdel‐Azim et al., 2011; Belewu et al., 2003; El‐Bordeny et al., 2015; Fouda, 2008). Moreover, growing animals fed on biologically treated roughages showed better daily gain (Allam et al., 2006), FCE, and pre‐slaughter live weight (El‐Marakby, 2003) compared to animals fed untreated roughages. In El‐Banna, El‐Manylam, et al. (2010), higher average daily gain (ADG) (19.72 vs. 21.5 g/day), pre‐slaughter weight (SW) (1885 vs. 1968 g), dressing percentage (DP) (59.9% vs. 61.6%), and lower FCE (4.56 vs. 4) were reported for rabbits fed on potato vines that were treated with *L. acidophilus* compared to rabbits fed untreated. Contrary to these results, the ADG of sheep fed SCB treated with brown rot fungi (*T. reesei F‐418*) was less than that of those fed untreated SCB (El‐Banna, Shalaby, et al., 2010), but FCE was increased in this study. However, few studies on the effects of biological treatment on animal performance have been conducted in Ethiopia (Mulgeta, 2015; Tibebu, 2019).

We hypothesized that feeding biologically treated SCB and RH resulted in different responses in terms of growth, carcass traits, and economic efficiency, which helped us to decide which feed produced the greatest results in terms of growth, carcass, and profitability. Therefore, the aim of this study was to evaluate the effects of *Trichoderma viride* (Tv) and effective microorganism (EM) treated SCB and RH in the concentrate mixture on weight change, carcass characteristics, and economic benefit of yearling rams.

## 2 MATERIALS AND METHODS

### 2.1 Study site

The experiment was performed at the goat farm of Haramaya University. Haramaya University is located at 9°26′N latitude and 42^o^3′E longitude. The study site is approximately located at 1980 m above sea level and 520 km east of the capital city, Addis Ababa. The mean annual rainfall in the study site is about 870 mm with a range of 560–1260 mm and the mean maximum and minimum temperatures are 23.4 and 8.25°C, respectively (Mishra et al., 2004).

### 2.2 Sources of experimental feed

Rice husk, used in this study, was obtained from Bench Sheko Zone of the Southwestern Ethiopia Regional (SWER) state, and SCB, and Molasses were obtained from Wonji Sugar Factory. However, other feed ingredients like maize grain, wheat bran, and salt were purchased from Haramaya town market; vitamin premix and noug seed cake were purchased from Addis Ababa. The collected samples of RH and SCB were ensiled with Tv and effective microorganisms (EM) and mixed with other feed ingredients.

### 2.3 Treatment preparation, experiment design, and layout

*Trichoderma viride* was obtained from Department of Plant Protection, School of Plant Sciences, University of Haramaya, Ethiopia. *Trichoderma viride* sample stored at 4°C was taken and cultured on Petri dish to activate the microorganisms. The Tv slants of 3 days old were inoculated for 7 days on Petri dish (9 cm) contained 25 mL potato dextrose agars (PDA). The 7 days culture was used to inoculate a plastic bottle contained the following substances per liter of distilled water: 4% molasses, 0.4% urea, 0.2% KH_2_PO4, and 0.03% MgSO4 (7H_2_O) per liter of water and the inoculum was stored at room temperature for 7 days. RH and SCB were inoculated by the solution to make spawn. Subsequently, untreated SCB and RHs were moistened approximately to 55% and immunized with 10% of the corresponding spawning fungi of SCB and RH (Abdel‐Azim et al., 2011). Treatment was took place in a clean house covered with a plastic sheet chemically sterile with Dettol and Formalin. After mixing spawns and feeds, it was covered with plastic sheet and left to ferment for 21 days; then the treated materials were opened and placed under the sun for 8 h/day at about 10% and packed and kept until used for experimental trials.

Sufficient quantities of an inert form of EM (EM‐1) packaged in plastic flasks were obtained from Weljijie PLC found in Debrezeit. Molasses was added and mixed with EM in equal proportion to initiate the multiplication and metabolism activities of the microorganism. The EM and molasses mixture was diluted with chlorine free water at a ratio of 18 to 1 per liter, respectively (Higa & Wididana, 2007). After stirring, the solution was kept in a 200 liters capacity barrel for 7 days to activate the EM solution. Then the EM solution was sprayed evenly on to the SCB and RHs at a ratio of 1 L of EM per 1 kg of dry matter feed, thoroughly mixed and packed in an airtight plastic bag with a capacity of 100 kg and stored in a large barrel of capacity 200–250 kg, covered with plastic sheet and kept at room temperature for 21 days for feeding.

Six experimental diets were prepared from the concentrate mix and the two roughages (RH and SCB). The experimental diets had forage to concentrate ratio of 50:50 as shown in Tables 1 and 2. The treatments were randomly allocated to individually house 36 yearling rams in a Randomized Complete Block Design (RCBD), then each treatment were repeated six times. The experimental diets were formulated rendering to recommendation set by National Research Council (NRC, 2007) energy and crude protein requirements for sheep growing at least at 50 g/day. The rations were offered in the form of total mixed diet as free choices with a rejection rate of 20%; accustomed every 4 days according to intake of each animal in previous day. The daily offer was consisted two meal times at 8:00 a.m. and 2:00 p.m. The quantity of experimental feed offer and refusal was documented daily for individual animal to determine the daily intake of each animal as a difference between two meals. Feed samples were collected from groups of prepared feed, but refusal were taken daily for each animal, measured and combined by animal. Samples of experimental feed offered and refused were collected and recorded during experimental period and sub‐samples of these feed were taken to evaluate the chemical composition.

**TABLE 1:** Chemical compositions (% DM basis) of feed ingredients.

Feed ingredients	DM (%)	Nutrients (% DM)				
		OM	CP	NDF	ADF	ADL
Maize grain	89.0	98.4	9.2	13.7	1.0	1.7
Noug seed cake	92.1	89.6	31.6	38.5	20.1	8.5
Wheat bran	90.8	94.5	15.3	48.7	15.4	3.2
Rice husks (untreated)	90.3	82.4	6.8	69.3	47.8	14.4
Rice husks (RH‐EM)	45.4	80.9	7.2	59.8	38.6	14.4
Rice husks (RH‐Tv)	90.5	79.1	10.6	55.7	35.4	12.7
Sugarcane bagasse (untreated)	90.7	94.7	2.4	88.6	63.8	15.7
Sugarcane bagasse (SCB‐EM)	36.5	92.9	3.4	83.9	58.7	14.4
Sugarcane bagasse (SCB‐Tv)	90.2	91.2	5.9	79.8	58.5	13.6

Abbreviations: ADF, Acid detergent fiber; CP, Crude protein; DM, Dry matter; NDF, Neutral detergent fiber; OM, Organic matter; RHEM, Rice husk treated with effective microorganism; RHTv, Rice husk treated with *Trichoderma viride*; SCBEM, Sugarcane bagasse effective microorganism; SCBTv, Sugarcane bagasse treated with *Trichoderma viride*.

**TABLE 2:** Ingredients (%) of experimental diets and chemical composition (% DM basis).

Ingredients (%)	Treatments					
	Rice husk (RH)			Sugarcane bagasse (SCB)		
	U	EM	Tv	U	EM	Tv
Untreated RH	50	‐	‐	‐	‐	‐
EM treated RH	‐	50	‐	‐	‐	‐
Tv treated RH	‐	‐	50		‐	‐
Untreated SCB	‐	‐	‐	50	‐	‐
EM treated SCB	‐	‐	‐	‐	50	‐
Tv treated SCB			‐	‐		50
Maize	11	11	11	11	11	11
Wheat bran	11	11	11	11	11	11
Noug seed cake	23.5	23.5	23.5	23.5	23.5	23.5
Molasses	3	3	3	3	3	3
Salt	1	1	1	1	1	1
Pre.mix	0.5	0.5	0.5	0.5	0.5	0.5
Chemical composition of diet (% DM basis)						
DM	89.6	66.6	89.5	89.8	61.1	89.6
OM	85.8	85.1	84.6	92.5	91.5	90.5
CP	13.7	13.8	15.6	11.4	11.9	13.2
NDF	50.3	45.7	43.7	60.2	57.6	55.7
ADF	30	25.8	24	38.1	35.6	35.7
ADL	9.7	9.5	8.8	10.2	9.6	9.3
ME^a^ MJ/kg DM	10.59	11.37	12.05	10.4	11.2	11.14
TDN^b^	59.84	63.00	64.34	54.00	55.62	55.55

Abbreviations: ADF, Acid detergent fiber; ADL, Acid detergent lignin; CP, Crude protein; DM, Dry matter; EM, effective microorganism; ME, Metabolizable energy; NDF, Neutral detergent fiber; OM, Organic matter; Tv, *Trichoderma viride*; U, Untreated.

^a^ ME = DOM × 0.016 (McDonald et al., 2010); DOM = digestible organic matter per kg of DM.

^b^ TDN = 82.38‐(ADF × 0.7515) (NRC,  2001); premix contained: 500,000 IU vitamin A, 100,000 IU vitamin D3, 100 mg vitamin E, 190 g Ca, 90 g Mg, 50 g Na, 2 g Mn, 3 g Fe, 0.3 g Cu, 3 g Zn, 0.1 g Co and 1 mg Se.

### 2.4 Animals and management

Locally recognized Ethiopian highland sheep breed found in the highlands of Eastern Hararghe was used. Mature males weighed about 31.6 kg, while females weighed about 26.5 kg (Shibabaw et al., 2014). For this study, a total of 36 yearling intact rams with an average initial body weight (IBW) of 18 ± 1.8 kg were purchased from the local market. The ages of local rams were estimated through observation of their dentition and information obtained from the owners. Rams were subsequently taken to Haramaya University, where they were quarantined for 3 weeks before receiving internal parasite treatments and being ear‐tagged for identification. The lambs were put in an individual pen furnished with feeding and watering equipment. Animals were weighed for two consecutive days, after overnight feed withdrawal, and the average weights were taken as an IBW. Fresh tap water was furnished in a bucket twice daily, and feed was supplied in a feeding trough.

### 2.5 Growth trial

The fattening study lasted for 90 days. The quantities of experimental feed offered and refused were recorded every day throughout the study period using a digital weighing balance with compassion of 0.02 kg. Animals had been weighed at an interval of 10 days after overnight feed withdrawal, and earlier than daily feed was offered using a hanging scale graduated in 0.2 kg intervals. The differences between final and initial weights were used to calculate total weight gain (TWG). ADG was determined as the difference between the final body weight (FBW) and IBW of rams divided by the number of feeding days. Animal FCE was calculated as the ratio of daily weight gain to daily DM intake.

### 2.6 Laboratory chemical analysis of feeds and meat samples

Chemical analysis of samples was done in the Haramaya University Animal Nutrition Laboratory. Demonstrative samples of meat, experimental feed, and refusal were dried at 60°C for 72 h. Dried samples were crushed using a laboratory mill to pass through a 1 mm screen and kept for subsequent analyses of dry matter (DM), crude protein (CP), ash (AOAC, 1990), acid detergent fiber (ADF), neutral detergent fiber (NDF), and acid detergent lignin (ADL) (Van Soest & Robertson, 1985). The CP was calculated as N × 6.25. Meat samples were taken from the areas of Longissimus dorsi muscle (LDM) at the 10th and 13th rib positions, chopped, thoroughly mixed, and frozen at −20°C until partially dried. [Correction added 5 October 2023; ‐200°C corrected to ‐20°C.] The frozen fresh samples were partially dried at 55°C for 72 h, packed in polyethylene bags, and stored in a refrigerator at 4°C pending proximate chemical analysis. [Correction added 5 October 2023; 550°C corrected to 55°C and 40°C corrected to 4°C.] About 2 g of partially dried samples were weighed into a pre‐weighed crucible dish, and dried in an oven at 105°C overnight to DM. [Correction added 5 October 2023; 1050°C corrected to 105°C.] Ash content was determined by burning the samples dried during moisture determination by incinerating in a muffle furnace at 600°C for 12 hours, cooling in desiccators, and weighing the incinerated sample (ash). Ash content was expressed as a percentage of the weight of ash to the weight of the dried sample. Nitrogen (N) was analyzed by the Kjeldahl method (AOAC, 1990), and crude protein was determined as N × 6.25. Soxhlet methods were used to extract crude fat (CF).

### 2.7 Carcass evaluation

All rams were fasted overnight, weighed, and slaughtered at the end of the experimental period. Animals were killed by severing their jugular vein and carotid artery with a knife. During the slaughter process, total edible offal components (TEOC) data were carefully recorded, including the weight of blood, heart, liver with gall bladder, tail, kidneys, empty gut, and fat (omental, intestinal, and kidney); and total non‐edible offal components (TNEOC) as the sum of the weight of the head, lung with trachea, skin, spleen, penis, testis, gut content, and feet. The difference between SW and gut content was used to calculate the empty body weight (EBW) of slaughtered animals. DP was determined as hot carcass weight (HCW) divided by SWB and/or HCW divided by EBW. Both the right and left rib‐eye area (REA) were cut between the 12th and 13th ribs perpendicular to the backbone to measure the cross‐section of the rib‐eye muscle. Rib‐eye muscle was traced first on transparency paper, then the left and right REM areas were traced on square paper, which was placed on the transparency, and the area of the squares (0.25 cm^2^ each) that fell within the traced area was counted, those that partially fell outside were estimated, and the average of the two sides was taken as the REM area.

### 2.8 Partial budget analysis

A profitability analysis was demonstrated to evaluate the profitability of feeding by‐products (SCB and RH) treated with EM and Tv incorporated into the concentrate mixture containing maize, noug seed cake, molasses, and salt to Hararghe highland sheep. The analysis was performed considering the main input costs, such as sheep prices, feed prices, and labor expenses. The average price was taken as the purchasing price of sheep for all treatments. The cost of feed treatments was estimated and added to the feed cost. The selling price of each animal was estimated by three experienced individuals involved in sheep trading in the area. The total return (TR) was taken as the difference between the average selling and purchase prices of each animal. The calculations for the following economic parameters were done according to Upton (1979), as follows:

NR = TR−TVC; MRR = ∆NR/∆TVC; Where NR = net return; TR = total return; TVC = total variable cost; MRR = marginal rate of return.

### 2.9 Statistical data analysis

Data collected on growth and carcass characteristics of sheep consumed on respective treatment diets were analyzed using SAS software 9.3 (SAS, 2014). Where there is a significant difference between means, Tukey honestly significance tests at *p* < .05 was considered as significantly difference among treatments to adjust the mean separation. The model employed was: *Y*_*ijkl*_ = *μ* + *A*_*i*_ + *B*_*j*_ + *C*_*l*_ + *B* × *C*_*ij*_ + *ε*_*ijkl*_; Where: *Y*_*ijkl*_ = the dependent variables, *μ* = The overall mean, *A*_*i*_ = The *i*th block, *B*_*j*_ = The *j*th feed type, *C*_*l*_ = The *l*th treatment method, *C* × *B* jl= The *j*lth interaction (between feed type and treatment method), *ε*_*ijkl*_ = random error.

## 3 RESULTS

### 3.1 Feed chemical compositions

The chemical compositions of the diets used in the current study are presented in Tables 1 and 2. Crude protein content of diets containing RH was higher than that of diets containing SCB. SCB containing diets had higher fiber components (ADF and NDF) compared to diets prepared from RH. Inclusion of biologically treated by‐products reduces dietary fiber components (NDF, ADF, and ADL), but CP and Ash contents were increased.

### 3.2 Performances of rams

Significantly (*p* < .05) higher FBW, TWG, ADG, and FCE were recorded for sheep consumed experimental diets containing RH compared to those consuming diets containing SCB (Table 3). Correspondingly, FBW, TWG, and ADG were also affected by biological treatments. Higher (*p* < .05) values of these parameters were obtained from sheep that consumed diets containing biologically treated by‐products compared to animals fed diets containing untreated by‐products. Nevertheless, there was no significant difference detected between EM and Tv. Values of dry matter intake (DMI) were significantly (*p* < .05) higher for rams consumed diet contained RH. Likewise, DMI was significantly (*p* < .05) increased for groups that consumed diets containing biologically treated by‐products.

**TABLE 3:** Growth parameters of rams consumed diet containing by‐products treated with *Trichoderma viride* and EM.

Parameters	Feed (F)			Treatments (T)				*p*‐value		
	RH	SCB	SEM	U	EM	Tv	SEM	F	T	F × T
IBW (kg)	18.1	18.1	0.52	18.1	18	18.2	0.64	.98	.99	.99
FBW (kg)	27.6^a^	22.9^b^	0.54	23.8^b^	26.2^a^	25.8^ab^	0.66	<.001	.04	.49
TWG (kg)	9.5^a^	4.7^b^	0.28	5.7^b^	8.1^a^	7.6^a^	0.34	<.001	<.001	.1
ADG (g/d)	105.9^a^	52.7^b^	3.1	62.9^b^	90.4^a^	84.5^a^	3.8	<.001	<.001	.1
DMI (g/h/day)	986.5^a^	679.6^b^	22.3	748.5^b^	890.7^a^	856.9^a^	27.3	<.001	.03	.01
DMI (% of BW)	3.60^a^	2.96^b^	0.06	3.11^b^	3.40^a^	3.28^a^	0.04	<.001	.04	.01
DMI (g/kg BW^0.75^)	82.30^a^	64.64^b^	1.50	68.77^b^	79.91^a^	74.74^a^	1.80	<.001	.01	.01
FCE	0.107^a^	0.076^b^	0.003	0.080^b^	0.100^a^	0.096^ab^	0.004	<.001	.001	.25

*Note*: Least square means of each parameter with different superscripts (a and b) within the same column are significantly different at *p* < .05.

Abbreviations: ADG, Average daily gain; BW, Body weight; DMI, Dry matter intake; EM, effective microorganism; FBW, Final body weight; FCE, Feed conversion efficiency (g dg/g DDMI); IBW, Initial body weight; RH, Rice husk; SCB, Sugarcane bagasse; SEM, Standard Error Mean; Tv, *Trichoderma viride*; TWG, Total weight gain; U, Untreated.

### 3.3 Carcass components

The results of the carcass parameters of Hararghe highland sheep are presented in Table 4. Slaughter body weight (SBW), EBW, HCW, DP, and REA were significantly (*p* < .05) higher for rams consumed experimental feed containing RH compared to sheep consumed SCB‐based diets. Biological treatment has an effect on the carcass components of animals. Sheep fed diets containing by‐products treated with EM and Tv had significantly (*p* < .05) higher SBW, HCW, and DP than sheep fed diets based on untreated roughages. However, there were no significant differences between EM and Tv in carcass parameters.

**TABLE 4:** Carcass components of rams fed diet containing RH or SCB treated with *Trichoderma viride* and EM.

Carcass parameter	Feed (F)			Treatments (T)				*p*‐value		
	RH	SCB	SEM	U	EM	Tv	SEM	F	T	F × T
Slaughter body weight (kg)	27.6^a^	22.8^b^	0.54	23.8^b^	26.2^a^	25.8^a^	0.66	<.001	.04	.5
Empty body weight (kg)	22.8^a^	19.1^b^	0.50	19.8	21.7	21.4	0.55	<.001	.06	.2
Hot carcass weight (kg)	12.4^a^	9.1^b^	0.32	9.7^b^	11.4^a^	11.1^a^	0.4	<.001	.01	.5
DP										
SBW basis	44.6^a^	39.9^b^	0.62	40.5^b^	43.4^a^	43.0^a^	0.76	<.001	.02	.6
EBW basis	54.5^a^	47.6^b^	0.9	49.0	52.5	51.9	1.1	<.001	.07	.6
REM area (cm^2^)	9.92^a^	7.48^b^	0.29	8.23	9.12	8.74	0.36	<.001	.22	.6

*Note*: Least square means of each parameter with different superscripts (a and b) within the same column are significantly different at *p* < .05.

Abbreviations: DP, Dressing percentage; EBW, empty body weight basis; EM, Effective microbes; HCW, Hot carcass weight; REMA, Rib eye muscle area; RH, Rice husk; SBW, slaughter body weight basis; SCB, Sugarcane bagasse; SEM, pooled standard error of mean; Tv, *Trichoderma viride*; U, Untreated.

### 3.4 Non‐carcass edible components

Weight of the blood, heart, and tongue were not affected (*p* > .05) by both feed type and biological treatments (Table 5). However, liver with gall bladder, kidney, and empty gut weights were significantly higher in sheep that consumed diets containing RH. Similarly, heart fat, and omentum and mesenteric fat were significantly higher in the sheep that consumed RH diets. Weight of total non‐carcass fat (TNCF) was significantly lower for sheep receiving diets containing SCB. Total edible offal (TEO) was significantly higher in the RH group than the SCB ones. Biological treatments had no significant effect on individual non‐carcass edible components, but total edible offal (TEO) was significantly (*p* < .05) higher for feed treated with *Trichoderma viride* (Tv).

**TABLE 5:** Edible offal components of Hararghe highland sheep fed with diet containing biologically treated RH or SCB.

Parameters	Feed (F)			Treatments (T)				*p*‐value		
	RH	SCB	SEM	U	EM	Tv	SEM	F	T	F × T
Blood (kg)	0.92	0.84	0.036	0.88	0.92	0.84	0.044	.08	.46	.92
Liver + gall bladder (kg)	0.36^a^	0.26^b^	0.011	0.3	0.3	0.33	0.014	<.001	.062	.35
Heart (kg)	0.07	0.07	0.002	0.07	0.07	0.07	0.003	.24	.26	.88
Kidney (kg)	0.07^a^	0.06^b^	0.002	0.06	0.06	0.07	0.003	<.001	.07	.57
Tail (kg)	1.06^a^	0.56^b^	0.043	0.67^b^	0.95^a^	0.81^ab^	0.008	<.001	.0032	.32
Empty gut (kg)	1.31^a^	1.16^b^	0.03	1.2	1.22	1.28	0.04	.001	.32	.16
Tongue (kg)	0.05	0.05	0.004	0.05	0.05	0.06	0.005	.94	.45	.87
Omenetal & M.fat	0.32^a^	0.20^b^	0.018	0.26	0.23	0.28	0.023	<.001	.17	.01
Heart & kidney fat (kg)	0.11^a^	0.084^b^	0.007	0.10	0.09	0.10	0.008	.008	.5	.01
TNCF (kg)	0.42^a^	0.28^b^	0.02	0.35	0.32	0.38	0.026	<.001	.18	.03
TEO (kg)	4.7^a^	3.6^b^	0.12	3.86^b^	4.16^ab^	4.46^a^	0.15	<.001	.023	.3

*Note*: Least square means of each parameter with different superscripts (a and b) within the same column are significantly different at *p* < .05.

Abbreviations: EM, Effective microbes; RH, Rice husk; SCB, Sugarcane bagasse; SEM, pooled standard error of mean; TEO, Total edible offal; TNCF, Total non‐carcass fat; Tv, *Trichoderma viride*; U, Untreated.

### 3.5 Non‐edible offal components

The result shows a significantly higher weight of head without tongue, testicles, spleen, esophagus, penis with fat, skin with legs, and gut content observed for sheep fed a RH‐based diet compared to a SCB based diet (Table 6). However, there was no significant difference in lung with trachea between the groups. Generally, total non‐edible offal (TNEO) was significantly higher in the RH group than the SCB group. Weight of the testicles, spleen, esophagus, skin with legs, and gut content are similar for sheep fed biologically treated roughage based diets. Likewise, significantly (*p* < .05) higher values of head without tongue and penis with fat were recorded for the feed treated with EM and lung with trachea for feed the treated with Tv.

**TABLE 6:** None edible offal components of Hararghe highland sheep fed with diet containing biologically treated RH or SCB.

Parameters	Feed (F)			Treatments (T)				*p*‐value		
	RH	SCB	SEM	U	EM	Tv	SEM	F	T	F × T
Head	1.60^a^	1.35^b^	0.03	1.38^b^	1.56^a^	1.46^ab^	0.037	<.001	.01	.36
Testicles (kg)	0.30^a^	0.25^b^	0.03	0.27	0.28	0.28	0.015	.003	.9	.52
Penis with fat (kg)	0.17^a^	0.10^b^	0.008	0.13^ab^	0.15^a^	0.12^b^	0.01	<.001	.04	.12
Spleen (kg)	0.03^a^	0.02^b^	0.0015	0.03	0.03	0.03	0.002	.003	.3	.07
Lung + trachea (kg)	0.25	0.24	0.01	0.22^b^	0.24^b^	0.27^a^	0.013	.49	.03	.05
Esophagus (kg)	0.03^b^	0.04^a^	0.004	0.03	0.04	0.04	0.005	.03	.2	.27
Skin with legs (kg)	3.84^a^	3.10^b^	0.095	3.26	3.63	3.51	0.11	<.001	.09	.88
Gut content (kg)	4.86^a^	3.87^b^	0.15	4.03	4.68	4.37	0.18	.005	.45	.49
TNEO (kg)	11.08^a^	8.80^b^	0.23	9.14	10.61	10.03	0.28	<.001	.09	.33

*Note*: Least square means of each parameter with different superscripts (a and b) within the same column are significantly different at *p* < .05.

Abbreviations: EM, Effective microbes; RH, Rice husk; SCB, Sugarcane bagasse; SEM pooled standard error of mean; TNEOC, Total non‐edible offal components; Tv, *Trichoderma viride*; U, Untreated.

### 3.6 Meat chemical compositions

Moisture, crude protein (CP), fat, and ash contents of meat were affected by feed type, and only ash was affected by biological treatments, whereas CP and fat were affected by interaction (Table 7). Significantly higher (*p* < .05) moisture content was observed in the meat of rams whose diets contained SCB than rams whose diets contained RH. The crude protein, fat, and ash concentrations were significantly higher (*p* < .05) for meat obtained from group fed diets containing RH. The effect of biological treatments on meat moisture, crude protein, and fat contents was not significant (*p* > .05), but higher meat ash content was recorded for group fed diets containing biologically treated (EM and Tv) by‐products. The crude protein content of meat obtained from rams consuming diets containing untreated RH and treated with effective microorganisms was significantly higher compared to meat obtained from groups consuming diets containing RH treated with Tv and either treated or untreated SCB. The ash content of meat obtained from rams consuming diets containing RH treated with Tv was higher compared to the group consuming diets containing untreated RH and RH treated with EM, and either treated or untreated SCB. But, both protein and ash contents of meat did not significantly vary between the groups consuming diets containing either treated or untreated (SCB).

**TABLE 7:** Meat proximate compositions (% DM) of rams fed diet containing biologically treated RH or SCB.

Variables	Feed (F)			Treatments (T)				*p*‐value		
	RH	SCB	SEM	U	EM	Tv	SEM	F	T	F × T
MO	70.69^b^	74.61^a^	0.6	72.45	72.2	73.3	0.7	<.001	.53	.1
CP	21.14^a^	20.34^b^	0.06	20.74	20.87	20.65	0.07	<.001	.12	.01
Fat	7.4^a^	6.05^b^	0.09	6.67	6.72	6.73	0.11	<.001	.93	.01
Ash	4.60^a^	3.90^b^	0.04	4.15^b^	4.30^a^	4.40^a^	0.05	<.001	.01	.5

*Note*: Least square means of each parameter with different superscripts (a and b) within the same column are significantly different at *p* < .05.

Abbreviations: CP, Crude protein; EM, effective microorganism; MO, Moisture; RH, Rice husk; SCB, Sugarcane bagasse; SEM, Standard Error Mean; Tv, *Trichoderma viride*; U, Untreated.

### 3.7 Partial budget analysis

Based on total variable cost (TVC), purchasing and selling prices of sheep, significantly (*p* < .05) highest net return obtained from rams consumed diet contained RH and biologically treated by‐products related to rams consumed untreated roughage‐based diets (Table 8). The differences in the net return among rams consuming diets containing RH and biologically treated by‐products are mainly due to higher body weight gain and good body conditions, which have contributed to an increased selling price.

**TABLE 8:** Economic return of rams fed diet containing biologically treated RH or SCB.

Variables	Feed (F)			Treatments (T)				*p*‐value		
	RH	SCB	SEM	U	EM	Tv	SEM	F	T	F × T
Purchasing cost (ETB)	820.00	820.00	36.91	820.00	820.00	820.00	45.20	1.00	1.00	1.00
Feed intake (kg)	985.52^a^	678.53^b^	22.32	7748.46^b^	890.71^a^	856.90^a^	27.33	<.001	.00	.00
Feed cost (ETB)	441.77^a^	275.03^b^	9.88	300.07^b^	893.61^a^	381.51^a^	12.10	<.001	.04	.12
Lab. cost (ETB)	52.00	52.00	0.00	52.00	52.00	52.00	0.00	<.001	<.001	.00
Total variable cost (ETB)	493.77^a^	327.03^b^	9.88	352.07^b^	445.61^a^	433.51^a^	12.10	<.001	<.001	.01
Selling price	1811.56^a^	1370.22^b^	46.79	1446.67^b^	1673.33^a^	1652.67^a^	57.31	<.001	.02	.47
TR (ETB)	991.56^a^	550.22^b^	30.53	626.67^b^	853.33^a^	832.67^a^	37.39	<.001	.00	.18
NR (ETB)	497.79^a^	223.19^b^	26.32	274.59^b^	407.72^a^	399.16^a^	32.24	<.001	.01	.62
∆NR	‐	274.6	‐	‐	133.13	124.57	‐	‐	‐	‐
∆TVC	‐	166.74	‐	‐	93.54	81.44	‐	‐	‐	‐
MRR	‐	1.64	‐	‐	1.42	1.53	‐	‐	‐	‐

*Note*: Least square means of each parameter with different superscripts (a and b) within the same column are significantly different at *p* < .05. Maize = 550 birr/Quintal, Wheat bran = 430 birr/Quintal, Noug cake = 750 birr/Quintal, Rice husk = 300 birr/Quintal, Sugarcane bagasse = 200 birr/Quintal, Molasses 2 birr/lit., Salt = 5 birr/kg, Premix = 50 birr/kg, EM = 20 birr/lit, Tv = 20 birr/lit.

Abbreviations: ∆NR, Change in net return; ∆TVC, Change in total variable cost; EM, Effective microbes; ETB, Ethiopian Birr; MRR, Marginal rate of return; NR, Net return; RH, Rice husk; SCB, Sugarcane bagasse; SEM, pooled standard error of mean; TR, Total return; Tv, *Trichoderma viride*; U, Untreated.

## 4 DISCUSSION

### 4.1 Feed chemical compositions

Variations in the chemical compositions of the experimental diets in the current study are due to differences in feed chemical composition and biological treatments. The chemical compositions of diets containing biologically treated by‐products were similar to those of earlier studies that showed decreased organic matter (OM), neutral detergent fiber (NDF), acid detergent fiber (ADF) and increased crude protein (CP) and ash (Mahrous et al., 2021; Omer et al., 2012). This might be due to the utilization of OM, NDF, and ADF as carbon sources during the incubation period by Tv and EM for their growth, which subsequently increases the decomposition of agro‐industrial by‐products (Mahrous et al., 2021). Higher CP contents in diets containing Tv treated RH and SCB might be related to the carbohydrate utilization by microorganisms, which in turn results in higher CP contents (Mahrous et al., 2021; Omer et al., 2012).

### 4.2 Performances of rams

The current study shows that the body weight change recorded for sheep fed the RH diet (9.5 kg) is higher than the body weight change recorded for sheep fed the SCB diet (4.7 kg). The greater weight of sheep fed a RH‐based diet compared to a SCB‐based diet can be ascribed to the increased protein intake, which donates more nitrogen to the rumen microorganisms (Van Soest, 1994). Higher nitrogen intake with optimum nonstructural carbohydrate can increases the microbial population and efficiency, which increases the breakdown fiber component and leads to an increase in feed intake. The better performances recorded in sheep fed a total mixed ration containing biologically treated by‐products treated was attributed to the degradation of cell wall structure, which leads to increase digestibility and protein content of the diets. This in line with the findings of various researcher who reported that incorporating biologically treated roughage in the diets of ruminant increases feed intake, improves digestibility, and increases FCE (Abdel‐Azim et al., 2011; Belewu et al., 2003; El‐Bordeny et al., 2015; Fouda, 2008). Moreover, Gado et al. (2006) found that goats consumed with biologically treated SCB had higher DMI and average body weight gain. Contrary to the present study, the ADG of sheep fed a diet based on SCB treated with brown rot fungi (Trichodermareessei F‐418) was less than that of sheep fed a diet based on untreated SCB (El‐Banna, Shalaby, et al., 2010), but FCE was increased for this group. Value of the present study also agrees with the value reported for the same breed at the maximum level of supplementation (Diba et al., 2015; Hirut et al., 2011; Kefyalew et al., 2015). Similarly, the result is comparable with the findings reported for Arsi‐Bale sheep fed a basal diet of hay and supplemented with 300 g DM of sole or a mixture of linseed meal and wheat bran (Abebe et al., 2010).

### 4.3 Carcass component

The higher carcass weight recorded for RH and biological treated diet might be ascribed to higher feed intake and greater nutrient digestibility, which promoted weight gain and tissue developments. Agreement to this result, Ebrahimi et al. (2007) reported that well‐formulated ration with 14.5% crude protein could be improved feed intake, live weight gain, hot carcass weight, and overall performance of sheep. The carcass weight (12.4 kg) observed for RH diet in the present study was comparable with the value (12.1 kg) reported for Ethiopian highland ram lambs fed two varieties of maize silage or Stover forms and supplemented with wheat bran and noug seed cake (Tesfaye et al., 2019). However, the value (9.1 kg) recorded for SCB diet is within the range of value (5.2–9.1 kg) reported for the same breed consumed different basal diet supplemented with different levels of concentrate (Diba et al., 2015; Hirut et al., 2011; Kefyalew et al., 2015; Tagaynesh, 2014).

The higher DP stated as percentage of slaughter body weight and EBW, in RH diet could be an attribute of the higher hot carcass weight compared to SCB. Biological treatment also increases DP from 40.5% to 43.4% these could be ascribed to higher feed intake and nutrient digestibility accordingly improve the live weight of animals. Value of current study is within a range (36.7%–45.2%) reported for other indigenous sheep breeds supplemented with different diets (Getnet et al., 2008; Jemal et al., 2005; Takele & Getachew, 2011).

Rib‐eye muscle area is an indirect measurement of body musculature and amount of lean meat in the carcass (Wolf et al., 1980). The Rib‐eye muscle area (9.92 cm^2^) observed for sheep fed RH diet is higher than the value reported for Hararghe highland sheep (3.7–8.4 cm^2^) consumed different concentrate level and basal diet (Diba et al., 2015; Hirut et al., 2011; Kefyalew et al., 2015). The Rib eye muscle area is positively correlated with SW (Park et al., 2002), which can be impacted by nutrition. Thus, the high protein and energy content of RH promote muscle tissue development and live weight gain. On contrary to this result, Ríos‐Rincón et al. (2014) reported that rib eye muscle area was not significantly affected with different diet consisting of different proportion of protein and energy. El‐Banna, El‐Manylam, et al. (2010) reported higher ADG (19.72 vs. 21.5 g/day), pre‐SW (1885 vs. 1968 g), DP (59.9% vs. 61.6%), and lower FCE (4.56 vs. 4) for rabbits fed on potato vines that were treated with *L. acidophilus* compared to rabbits fed untreated.

### 4.4 Non‐carcass components

The feeds the animals consume not only affect the mass of live body weight and carcass but also the mass of viscera organs (Lawrence & Fowler, 1998). In the present study, higher value of liver with gallbladder, kidney, empty gut, TNCF, and total edible non‐carcass components were obtained for rams consumed diet containing RH as related to rams consumed diet contained SCB; might be due to high energy and protein content of the RH‐based diet. Archimède et al. (2008) stated that the amount of fat deposit is highly correlated with plane of nutrition or energy content of the diet and appropriate dietary energy to protein combinations. On the other hand, the higher liver weight for this group might be related to the storage of reserve substances such as glycogen as described by Lawrence and Fowler (1998).

Higher values of total edible offals were recorded for rams fed RH diet and Tv‐treated diet. The TEO recorded (3.6%–4.7%) was higher than the value obtained by Tsehay (2012) in Hararghe highland sheep (2.7%–3.2%) fed a basal diet of natural pasture hay and supplemented with graded levels of onion leaves as a substitute for wheat bran in concentrate mixture. But, it falls within a range (2.22–4.19 kg) reported by other authors (Diba et al., 2015; Hirut et al., 2011).The total non‐edible offal (8.80–11.08 kg) recorded for this study was slightly higher than value (8.09–9.43 kg) reported for the same breed (Diba et al., 2015; Hirut et al., 2011). Generally, rams consumed with diet contained RH and biologically treated by‐products recorded higher weights of edible and non‐edible offal than sheep feed with SCB and untreated by‐products. Therefore, feeding of Hararghe highland rams with concentrate mixture containing RH and biological treated by‐products could produce higher offal than group fed diet containing SCB and untreated by‐products.

### 4.5 Meat chemical compositions

The differences in moisture, crude protein, fat, and ash content of meat may be due to the variations in the nutrient composition of the feed consumed by animals. Values of moisture, fat, and ash contents were lower than the values reported by Shashie et al. (2018) and comparable with the values of the same breed reported by Diba et al. (2015). The lower moisture and higher fat contents recorded for meat obtained from sheep consumed diets contained RH were due to higher dietary energy and protein intake, as dietary energy and protein intake have a negative effect on moisture and a positive effect on fat content of the meat (Claffey et al., 2018). Values of meat fat content beyond optimum levels negatively affect the flavor of meat due to production of volatiles (Melton, 1990). This could be increase blood cholesterol levels, which is a risk factor for cardiovascular diseases. However, optimum fat content improves the quality (juiciness, flavor, and texture) of meat. The values (6.2%–7.8%) of meat fat content of all rams in the current study is slightly higher than the optimum value (5%) recommended by Hui et al. (2001) and less than the values (9.8%–10.9%) reported by Shashie et al. (2018) for rams of different Ethiopian breeds. However, Moloney and Teagasc (2002) and Chan (1995, 1996) reported that values of 5%–9.4% of ram meat fat content are normal for consumption in human diets. Higher crude protein content of meat from rams consumed diets containing RH than group fed diets containing SCB could be due to higher protein to energy ratio intake. Furthermore, high protein and energy ratio intake can enhance the production of rumen microbes, which produce high‐quality microbial protein available for lower intestinal digestion and absorption. Thus, enhances the development of lean muscle in meat, which is the preferred trait in mutton (Diba et al., 2015). The higher ash content of meat from group received diet contained RH than sheep received diet contained SCB might be due to higher ash content of diets made from RH. The high net return obtained from rams consumed diet containing biologically treated RH or SCB is due to the greater body weight gain, good body condition as result of higher nutrient intake compared to rams consumed diets containing untreated RH or SCB.

## 5 CONCLUSIONS

The result in the current study showed that rams consumed with diet contained RH performed better in feed intake, body weight change, carcass yield, and profitability. Likewise, live weight changes, carcass yield, and profitability were highest for rams consumed diet containing biologically treated by‐products. Hence, it can be concluded that total mixed ration containing either treated or untreated RH more economically feasible than rams consumed either treated or untreated SCB. However, further study will be needed to determine the levels of inclusion of either treated or untreated SCB in ruminant ration in order to get maximum benefit.

## AUTHOR CONTRIBUTIONS

**Regasa Begna:** Conceptualization (equal); data curation (equal); formal analysis (equal); investigation (equal); methodology (equal); resources (equal); software (equal); validation (equal); writing – original draft (equal); writing – review and editing (equal). **Mengistu Urge:** Conceptualization (equal); project administration (equal); resources (equal); supervision (equal); visualization (equal); writing – original draft (equal); writing – review and editing (equal). **Tegene Negesse:** Conceptualization (equal); methodology (equal); project administration (equal); supervision (equal); writing – review and editing (equal). **Getachew Animut:** Conceptualization (equal); methodology (equal); supervision (equal); writing – review and editing (equal).

## CONFLICT OF INTEREST STATEMENT

The authors declare no conflicts of interest.

## ETHICS STATEMENT

Animal care and ethical issue were careful evaluated and approved the experiment (1956ET‐12/2017) by Haramaya University, College of Agriculture and Environmental Science ethics Committee. Directive 2010/63/EU of the European Union guidelines (2010) concerning the treatment and use of animals in research and development purposes were employed.

## Data Availability

Data used and analyzed for this study available from the corresponding author on reasonable request.
